# Saliva Alternative to Upper Respiratory Swabs for SARS-CoV-2 Diagnosis

**DOI:** 10.3201/eid2611.203283

**Published:** 2020-11

**Authors:** Rachel L. Byrne, Grant A. Kay, Konstantina Kontogianni, Ghaith Aljayyoussi, Lottie Brown, Andrea M. Collins, Luis E. Cuevas, Daniela M. Ferreira, Alice J. Fraser, Gala Garrod, Helen Hill, Grant L. Hughes, Stefanie Menzies, Elena Mitsi, Sophie I. Owen, Edward I. Patterson, Christopher T. Williams, Angela Hyder-Wright, Emily R. Adams, Ana I. Cubas-Atienzar

**Affiliations:** Liverpool School of Tropical Medicine, Liverpool, UK (R.L. Byrne, G.A. Kay, K. Kontogianni, G. Aljayyoussi, L. Brown, A.M. Collins, L.E. Cuevas, D.M. Ferreira, A.J. Fraser, G. Garrod, H. Hill, G.L. Hughes, S. Menzies, E. Mitsi, S.I. Owen, E.I. Patterson, C.T. Williams, A. Hyder-Wright, E.R. Adams, A.I. Cubas-Atienzar);; National Institute for Health Research, Leeds, UK (A.M. Collins, H. Hill, A. Hyder-Wright);; Liverpool University Hospitals National Health Services Foundation Trust, Liverpool (A.M. Collins, H. Hill, A. Hyder-Wright)

**Keywords:** SARS-CoV-2, COVID-19, RT-qPCR, swabs, saliva, respiratory infections, severe acute respiratory syndrome coronavirus 2, 2019 novel coronavirus disease, coronavirus disease, zoonoses, viruses, coronavirus, diagnosis, testing, upper respiratory swab samples

## Abstract

PCR of upper respiratory specimens is the diagnostic standard for severe acute respiratory syndrome coronavirus 2 infection. However, saliva sampling is an easy alternative to nasal and throat swabbing. We found similar viral loads in saliva samples and in nasal and throat swab samples from 110 patients with coronavirus disease.

Quantitative reverse transcription PCR (qRT-PCR) is the diagnostic standard for severe acute respiratory syndrome coronavirus 2 (SARS-CoV-2), the causative agent of coronavirus disease (COVID-19) (*1*). Testing usually is conducted on upper respiratory specimens collected using swabs (*1*–*3*). However, this method requires multiple samples and has a low sensitivity (*4*). Swab sampling can cause patients to cough or sneeze, uncomfortable reactions that might also increase transmission risks to healthcare workers (A. Wyllie et al., unpub. data, https://doi.org/10.1101/2020.04.16.20067835). Sampling technique proficiency also varies, especially during self-sampling (A. Wyllie et al., unpub. data, https://doi.org/10.1101/2020.04.16.20067835), which can result in false negatives. Furthermore, shortages of swabs, transport media, and personal protective equipment limit healthcare capacity to conduct SARS-CoV-2 tests that rely on swab sampling.

Saliva sampling is a noninvasive alternative to upper respiratory swabbing. We compared paired self-collected saliva samples with healthcare worker–collected nasal and throat swab specimens from 110 patients with suspected SARS-CoV-2 infection. This analysis was part of a prospective study (Facilitating a SARS CoV-2 Test for Rapid Triage) at the Royal Liverpool University and Aintree University Hospitals (Liverpool, UK). We recruited participants who had provided written informed consent and had COVID-19 symptoms. The National Health Service Research Ethics Committee (20/SC/0169) approved the study under Integrated Research Application System no. 282147.

Within 24 hours after patient consent, we collected nasal and throat swab specimens containing 1.0 mL of Amies transport medium (COPAN Diagnostics, https://www.copanusa.com). We also asked participants to funnel their saliva into a sterile cryotube (SARSTEDT, https://www.sarstedt.com). We immediately extracted RNA from the swab samples; we stored saliva samples at –80°C until processing. We extracted viral RNA using the QIAamp Viral RNA Mini Kit (QIAGEN, https://www.qiagen.com) and tested 8 μL of extracted RNA using the genesig Real-Time Coronavirus COVID-19 PCR (genesig, https://www.genesig.com). We quantified viral loads using the manufacturer’s positive control (1.67 × 10^5^ copies/µL) as reference.

Of the 110 adults recruited from April through June 2020, a total of 61 (55.5%) were women. Most participants were hospitalized; 21 (19.1%) were discharged to home directly from the emergency department. Overall, 12 (10.9%) saliva and 14 (12.7%) nasal and throat swab specimens of 110 paired samples tested positive for SARS-CoV-2 RNA. Viral loads for all samples ranged from 36 to 3.3 × 10^6^ copies/mL. Overall viral loads were similar among all positive samples (Figure).

**Figure:**
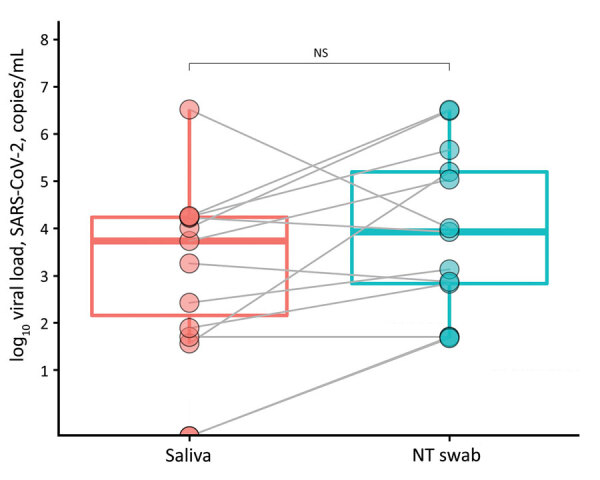
Viral load (copies/mL) of SARS-CoV-2 RNA recovered from paired saliva samples and nasal and throat swab specimens from 14 patients with coronavirus disease, United Kingdom, 2020. Viral loads are shown on a logarithmic scale. NS, not significant; NT, nasal and throat; SARS-CoV-2, severe acute respiratory syndrome coronavirus 2.

Insignificant viral load discrepancies existed among all positive samples (p = 0.1955 by Wilcoxon signed-rank paired test). Two patients tested positive (<10 copies/mL) on nasal and throat swab samples and negative on saliva samples; the discrepancies might have resulted from the different processing times of the 2 specimens because freeze-thawing can reduce the stability of RNA (*5*,*6*).

Saliva sampling can improve SARS-CoV-2 diagnostic techniques. Saliva samples are easier to collect than nasal and throat samples; the technique is noninvasive, presumably preferred by the participant, and does not require sampling proficiency. In addition, saliva sampling does not require swabs and transport media, which have limited availability during the pandemic. Our technique uses a funnel which, although helpful, might not be necessary for sample collection. Our study focused on symptomatic hospitalized participants; further research is needed on saliva sampling for patients with mild and asymptomatic SARS-CoV-2 infection.

Further studies should document the effects of storage and transport on RNA and viral loads. Rapid processing of saliva samples might benefit patients in low- and middle-income countries, where the pandemic is still accelerating and swab availability is limited (*7*). Furthermore, high-income countries can establish a cold chain for sample transportation. A cold chain could enable home sampling and screening of children who have rejected swabbing. It could also streamline research studies that require repeat sampling.

As rates of SARS-CoV-2 infection increase, we must continue to investigate efficient diagnostic strategies. Easy and effective diagnostic techniques, such as saliva sampling, should be evaluated in certified clinical laboratories.
